# Possible involvement of oxytocin in modulating the stress response in lactating dairy cows

**DOI:** 10.3389/fpsyg.2014.00951

**Published:** 2014-09-02

**Authors:** Mhairi A. Sutherland, Mattie Tops

**Affiliations:** ^1^AgResearch Ltd, Ruakura Research CentreHamilton, New Zealand; ^2^Department of Clinical Psychology, VU University of AmsterdamAmsterdam, Netherlands

**Keywords:** oxytocin, cortisol, heart rate, stress, habituation, novelty, dairy cows

## Abstract

Oxytocin can attenuate the physiological and behavioral response to stress in animals. In this study we investigated the relationship between plasma oxytocin concentrations and the behavioral and physiological response of dairy cows to a repeated psychological stressor (novel environment). Twenty lactating multi-parous dairy cows were milked in a familiar milking parlor and in a novel environment. Blood samples were collected before and after milking in the familiar parlor (baseline) and on the second and fifth day in the novel parlor to measure plasma cortisol and oxytocin concentrations. Heart rate was recorded on all cows during milking in the familiar and novel environment. On all test days, the behavioral response of cows to milk cluster attachment was scored. On day 2 in the novel parlor, the oxytocin response, cortisol concentrations and heart rate were greater, and heart rate variability was lower than baseline values recorded in the familiar parlor. The results from this study suggest that oxytocin release is increased in response to exposure to a psychological stressor (novel environment) and that cows adapt to this stressor over time. After initial suppression, oxytocin levels increased over days of milking in a novel environment, whereas indicators of stress simultaneously decreased. Furthermore, the oxytocin increase was associated with habituation of the cortisol response in anticipation of milking in a novel environment, suggesting that oxytocin may be involved in habituation to a novel environment in dairy cows. This mechanism of habituation to novel environments may reflect an association between oxytocin and a “familiarization-habituation response” to repeated exposure to an initially novel environment that has previously been reported in humans.

## INTRODUCTION

Oxytocin is a neuropeptide that is released in response to a variety of physical and psychological challenges (Neumann, 2002). In rodents, the release of oxytocin into the peripheral circulation in response to a variety of stressors is well established (Hashiguchi et al., 1997; Windle et al., 1997; Amico et al., 2004). Studies have also shown that oxytocin can attenuate activation of the hypothalamic-pituitary-adrenal axis and behavioral response to stress in rodents (Windle et al., 1997; Amico et al., 2004). Furthermore, oxytocin has been linked with habituation of physiological responses (Kéri and Kiss, 2011). Moreover, in rodents oxytocin promotes social proximity-seeking in response to threat (Bowen and McGregor, 2014).

Dairy cattle can be exposed to a range of physical and psychological challenges throughout their lives including exposure to a novel environment (milking parlor), unfamiliar stock people and unfamiliar conspecifics, which can potentially elicit a stress response (Rushen et al., 2001; Van Reenen et al., 2002; Sutherland et al., 2012). In conjunction with elevated cortisol concentrations and heart rate, exposure to a novel environment was found to increase plasma oxytocin levels in dairy cows (Sutherland et al., 2012). Furthermore, Yayou et al. (2014) found that basal oxytocin levels in dairy calves were negatively associated with personality traits reflecting a response in the form of escape from novel environments in the open-field test. Therefore, it appears as though oxytocin is released in response to stress and can attenuate the behavioral response to a novel environment in dairy cattle.

Contrary to our previous finding that oxytocin levels in dairy cows increased during milking in a novel compared to a familiar environment (Sutherland et al., 2012), older studies reported that oxytocin levels were decreased during the first milking in a novel compared to a familiar environment (Bruckmaier et al., 1993, 1996; Macuhová et al., 2002). Those older studies also found that during subsequent visits to the unfamiliar environment oxytocin release steadily increased and returned toward the control level (Bruckmaier et al., 1996; Macuhová et al., 2002). The studies by Bruckmaier et al. (1996) and those of Sutherland et al. (2012) differed in that the former involved hormonal measurement from the first milking in a completely novel environment (the cows were transported to, and milked in, the operating theater of a research station), whereas in the latter study hormones were measured starting from the second day of milking in an environment in which only the milking parlor was novel (a herringbone milking parlor instead of the familiar rotary milking parlor). The inhibition and gradual return to control levels of oxytocin in a novel environment suggest that oxytocin may initially be suppressed when cows are experiencing high levels of stress (Bruckmaier et al., 1993, 1996; Macuhová et al., 2002). Stress was indicated by cortisol increases before milking that were larger in the novel than in the familiar environment (Bruckmaier et al., 1993). By contrast, oxytocin responses in a novel environment that is less stressful or more permissive for social coping (Sutherland et al., 2012) may be involved in coping mechanisms that facilitate habituation of stress responses.

Recent evidence suggests that oxytocin may be involved in social familiarity-induced anxiolysis (Tops et al., 2013), a complex behavior involving the integration of social cues of familiarity with contextual and emotional information to regulate anxiety-like behavior (Lungwitz et al., 2014). We recently reported salivary oxytocin responses to a novel social context in female human subjects (Tops et al., 2013). Oxytocin levels were related to both increased trust and negative mood. Moreover, when the subjects were again confronted with the same social context weeks later, those subjects that showed high trust and oxytocin at the first confrontation, now showed high trust and decreased (habituated) oxytocin levels. High initial oxytocin levels followed by habituation were associated with an increase in calmness from the first to the second confrontation. The results are consistent with a role of oxytocin-related social stress coping in the habituation of stress responses by promoting familiarization with novel social contexts (a “familiarization-habituation response”).

Habituation to repetitive challenges is an important mechanism to enable individuals to cope with their environment. However, currently little is known about the association between oxytocin and habituation to stress in humans or animals. A ready-made opportunity to learn more about the familiarization-habituation response is by turning to available studies of responses to novel compared to familiar environments and oxytocin levels that have been performed with dairy cows. For this purpose, we will reanalyze data from the recent study that reported that oxytocin levels in dairy cows increased during milking in a novel compared to a familiar environment (Sutherland et al., 2012). Therefore, in this study we investigated dynamics over time and the relationship between plasma oxytocin, plasma cortisol, heart rate and behavior in response to a repeated psychological stressor (novel environment) in dairy cows. We investigated whether oxytocin levels were initially decreased in a novel milking environment before milking. By calculating correlation coefficients between initial oxytocin and cortisol levels, we investigated whether initial suppression of oxytocin in a novel milking environment was shown by cows who displayed higher distress and/or anticipatory cortisol increases before milking. Moreover, also through correlational analyses, we tested whether cows who had a greater oxytocin response to milking in a novel environment subsequently showed signs of habituation of plasma cortisol, heart rate and behavior.

## MATERIALS AND METHODS

### ANIMALS

This study was conducted on a research farm, in South Waikato, New Zealand between April and May 2010. All procedures involving animals were approved by the AgResearch Ruakura Animal Ethics Committee, under the New Zealand Animal Welfare Act 1999. Results from this study have been published previously in Sutherland et al. (2012). The previously published analysis investigated the effect of temperament (defined based on exit time from a restraint device) on behavior, physiology and milk production of the cows in a familiar and novel milking environment. Here, we collapsed data over temperaments and focus on the plasma oxytocin response to novelty and its potential role in stress coping by comparing it to other stress-responsive measures. Of the results reported here, only the general increase in oxytocin levels during milking in a novel compared to familiar environment overlaps with results reported previously (Sutherland et al., 2012).

Twenty, multi-parous, pregnant, Friesian-cross dairy cows were selected for this study. Cows were on average five (5.1 ± 2.61) years of age. This study was conducted during late lactation (average of 268 days in milk) and all cows were milked once a day only.

### GENERAL EXPERIMENTAL DESIGN AND PROCEDURE

Behavior and physiology data were collected over a 5 day period during milking when cows were milked according to normal practice in a familiar rotary parlor. The data collected during this period was used to provide baseline behavior and physiology values.

The behavioral response of cows to milking was assessed daily using a flinch, step, and/or kick (FSK) score. During attachment of the milking cluster, cows were given an overall score based on the performance of FSK behaviors using a 4-point scale: 1 = no hind foot movement, cow may flinch, shiver or do nothing at all; 2 = hind leg lifted no higher than 20 cm, step or shuﬄe of a hind leg; 3 = hind leg lifted higher than 20 cm, step or forward kick of a hind leg; 4 = backward kick of hind leg.

**FIGURE 1:**
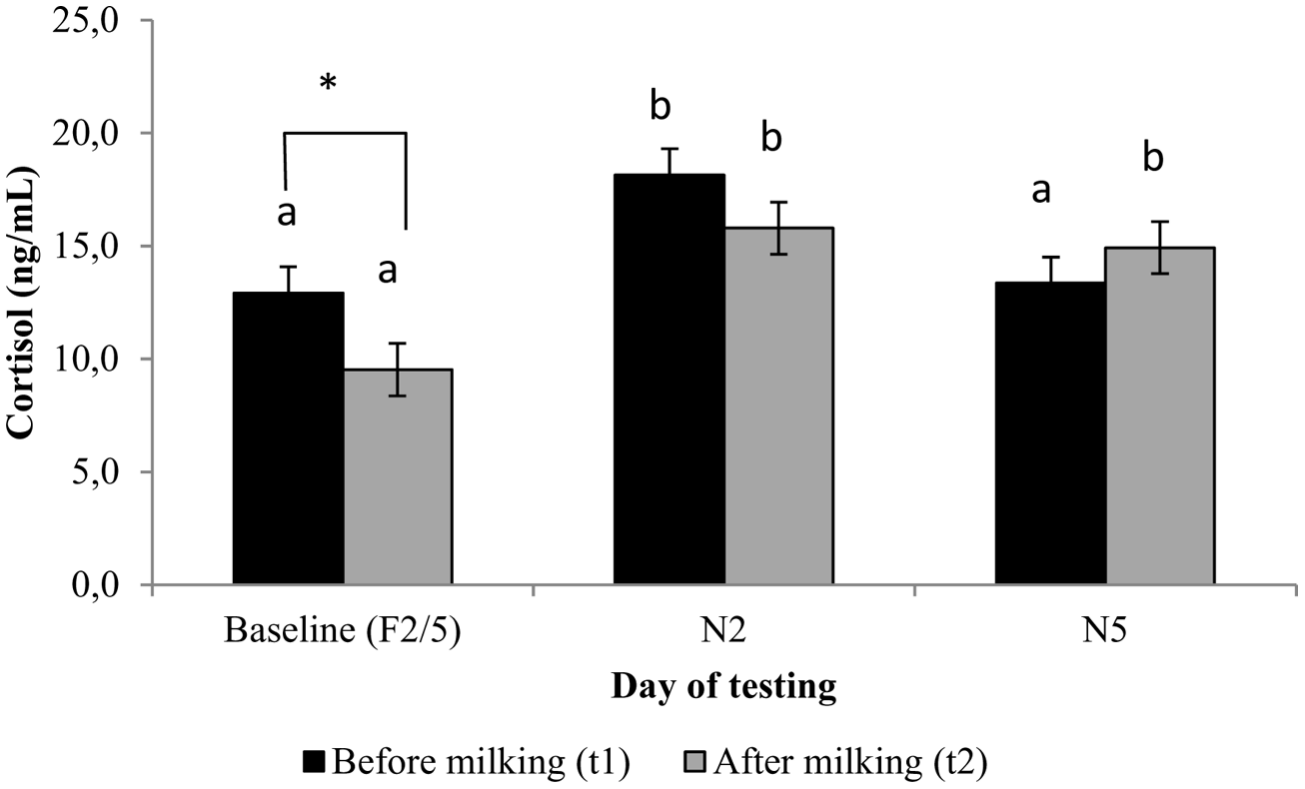
**Serum cortisol concentrations (ng/mL; least square means ± *SEM*) of cows (*n* = 20) before and after being milked in a familiar (Baseline) and novel milking environment (N2 and N5).** ^ab^Within sample collection time, least square means with different superscripts differ at *p* < 0.05. Means accompanied by an * differ at *p* < 0.05.

Blood samples were collected from the coccygeal vessels in the tail immediately before (t1) and after (t2) milking on the second and fifth day of milking during the baseline period for analysis of cortisol and oxytocin levels. To obtain baseline (familiar environment) values, the levels obtained from sampling on day 2 and 5 were averaged (“F2/5”). This resulted in six measurement “time points” for each hormonal variable: two baseline time points (F2/5t1, F2/5t2) and four time points in the novel parlor (N2t1, N2t2, N5t1, N5t2; see **Figure 1**). Prior to milking, all experimental cows were brought into a holding area and restrained as a group. Multiple people simultaneously collected blood samples from all cows to minimize the restraint time. Immediately after exiting the milking parlor after milking cows were moved to a holding area and the post-milking blood sample was collected. Blood collection took less than 30 s per cow. Blood was collected into uncoated and heparin-coated vacutainers (BD, Franklin Drive, Bridgeton, NJ, USA). Heparinized blood samples were placed immediately on ice and then centrifuged for 12 min at 3000 rpm at 4°C. Serum samples were left to clot for at least 2 h at room temperature prior to centrifugation. Serum and plasma samples were aspirated and stored at -20°C for future analysis. Serum samples were analyzed for cortisol concentrations using a commercially available radio-immunoassay kit (Siemens Cortisol-A-Count Kit, Cruinn Diagnostics Ltd., Dublin, Ireland). Plasma concentration of oxytocin was determined by ELISA (Assay Designs, Ann Arbor, MI, USA) according to the manufacturer’s instructions.

Heart rate was measured on each cow either during the first and third day of milking or the second and fourth day of milking, as it was not logistically possible to measure heart rate on all cows on the same days. Continuous interbeat intervals (IBI) were recorded during the 30 min period prior to milking and during milking using Polar heart rate monitors (S810i, Polar Electro Oy, Helsinki, Finland). Two days prior to testing, cows were restrained in a crush and silver wire electrodes (AS 826–32, Cooper Wire, Chatworth, CA, USA) were inserted subcutaneously behind the shoulder (distal to the withers) and directly behind the front leg near the brisket to improve conductivity of the heart rate monitors. Hair was clipped and the skin disinfected prior to wire insertion. The wire was threaded through an 18 g needle which was gently pushed through a fold of skin. The wire was then pushed through the shaft of the needle and the needle removed, leaving the wire in place and the ends were then joined to form a loop. These wires remained in the cows over the course of the study. When not in use the wires were protected by a Velcro patch placed over the exposed wire loop and covered by an elastic bandage and a surcingle around the animals’ girth. On the day of testing, the heart rate monitor leads were attached to the wire loops with alligator clips. Once the heart rate monitor was attached, the bandage and surcingle were replaced to protect the monitor during testing. After recording was completed, heart rate data were downloaded to a computer for analysis. Heart rate (beats/min) and the time domain parameter of heart rate variability (HRV), root mean square of successive R–R interval differences (rMSSD), were calculated from the IBI. Baseline heart rates were analyzed for 5 min while the cows were standing in the holding yards prior to entering the milking parlor and for the period they were being milked. For rMSSD analysis, short segments of data containing 256 beats were examined from a 5 min recording period as the cows stood in the holding yards before entering the milking parlor and for the period they were being milked. Before analysis, a correction function within the Polar software (Polar Precision Performance Software, version 4.03), set on default parameters, was used to correct for any artifacts and only data with an error rate of less than 5% were included in the analysis.

The week following collection of baseline behavior and physiology data, cows were milked once a day in a novel environment over a 5 day period. The novel environment was a herringbone milking parlor. The herringbone milking parlor was unfamiliar to all animals prior to testing. The herringbone parlor was situated next to the rotary milking parlor, so only the milking parlor was novel to the cows. The holding yards and stock handlers were the same as during the baseline testing period. Data were collected immediately before (t1) and after (t2) milking on the second (N2) and fifth (N5) day of milking during the test period in the same manner as during the baseline testing period in the familiar environment.

### STATISTICAL ANALYSIS

Data were subjected to analysis of variance using the mixed model procedure of SAS version 9.1 (SAS Inst., Inc., Cary, NC, USA). All data were tested for evidence of departures from the assumptions of both normality and homogeneous variance using residual diagnostic plots using the univariate procedure in SAS. Data lacking normality and transformed logarithmically included, cortisol and HRV. All analyses were fitted for treatment (milking environment) and day comparisons and treatment by day interactions. The model had a repeated structure on time allowing incorporation of heterogeneity of variances across time. Pearson correlation coefficients were calculated in SAS. Baseline heart rate and HRV data were defined as the average over a 5 min period prior to milking. Heart rate and HRV during milking were averaged over the first 5 min after the cow had entered the bale of the milking parlor. For clarity, we will refer to the first time in a five-day period that heart rate was measured as the day 2 measure, and the second time it was measured as the day 5 measure. Statistical significance was determined at *p* < 0.05 and trends were determined between *p* > 0.05 and *p* < 0.10.

## RESULTS

For mean (SE) behavior, HR, and HRV during milking, and cortisol and oxytocin change during milking, and significant differences between days, see **Table 1**. Because the previous studies discussed in the introduction suggested that milking in a novel environment may elicit separate processes of (1) initially high anticipatory stress responses involving cortisol, (2) initial suppression of oxytocin levels, and (3) a subsequently developing oxytocin response involved in habituation of responding to the novel environment, we will present analyses aimed at extracting each of those three processes in separate subsections.

**Table 1:** Least square mean (*SD*) behavioral and physiological response of cows milked in a familiar (Baseline) and a novel environment.

	Familiar environment		Novel environment				
Measure	Baseline (F2/5)	*SE*	Day 2 (N2)	*SE*	Day 5 (N5)	pSE*	*p*-Value
Flinch, step, kick score	1.5	0.14	1.8	0.19	1.5	0.19	0.339
Cortisol response (ng/mL)	-2.2^a^	1.15	-2.0^a^	1.79	1.8^b^	1.30	0.003
Oxytocin response (pg/mL)	25.8^a^	38.11	200.9^b^	53.22	125.6^ab^	53.22	0.027
Heart rate during milking (beats/min)	61.2^a^	1.54	68.7^b^	1.62	64.1^c^	1.73	0.001
HRV during milking (ms)	35.8^a^	6.16	30.8^b^	6.44	33.5^b^	6.03	0.006

^abc^Superscripts with different letters are different at p < 0.05. HRV, heart rate variability. p-Values of the treatment (milking environment) by day interactions are presented. *Pooled standard errors.

### ANTICIPATORY STRESS AS INDICATED BY CORTISOL, HR, AND HRV

#### Cortisol

For the mean cortisol levels at t1 and t2 on the different days, see **Figure 1**. On F2/5 results were consistent with an anticipatory cortisol response before milking, as F2/5t1 cortisol levels were higher than F2/5 t2 levels [*F*(2,146) = 3.31, *p* = 0.039]. Cortisol levels were higher at N2t1 than at F2/5t1 [*F*(2,146) = 7.49, *p* = 0.001] and N5t1 [*F*(2,146) = 5.32, *p* = 0.006], indicating that the anticipatory cortisol response was increased on N2. On N5, t1 cortisol returned to similar levels as on F2/5, but on N5 there was no decrease in cortisol from t1 to t2. Furthermore, t1 cortisol levels were positively correlated with HR during milking on F2/5 (*r* = 0.47, *p* = 0.035) and N2 (*r* = 0.64, *p* = 0.014) but not N5 (*r* = 0.14, *p* > 0.5). Note that in half of the cows, HR was measured on day 1 instead of day 2, and all cows were measured on day 3 or day 4 instead of day 5. This suggests that individual differences in stress levels in the early phase of novelty (days 1 and 2) related to higher cortisol levels and HR, and it is feasible that anticipatory stress before milking on day 2 (cortisol) related similarly to stress level during milking on day 1 and on day 2. However, in the later phase (days 3–5) the difference between the timing of measures and the variation in measurement times and individual differences in habituation of stress responses was likely to be too large for correlation between cortisol and HR.

#### Heart rate/Heart rate variability

On N2 HR during milking was higher than on F2/5 [*F*(2,57) = 14.19, *p* = 0.001] and on N5 [*F*(2,57) = 5.22, *p* = 0.008], while on N5 HR was still higher than on F2/5 [*F*(2,57) = 3.83, *p* = 0.028]. Similarly, on N2 HRV during milking was lower than on F2/5 [*F*(2,60) = 3.06, *p* = 0.055], while on N5 HRV was still lower than on F2/5 [*F*(2,60) = 4.58, *p* = 0.014]. Increased heart rate during milking was associated with a greater behavioral response to milk cluster attachment in a novel environment (*r* = 0.49, *p* = 0.033). The results of HR and HRV suggest that cows were experiencing higher levels of stress on N2 and lower levels on F2/5.

### INITIAL INHIBITION OF OXYTOCIN LEVELS BY STRESS

**FIGURE 2:**
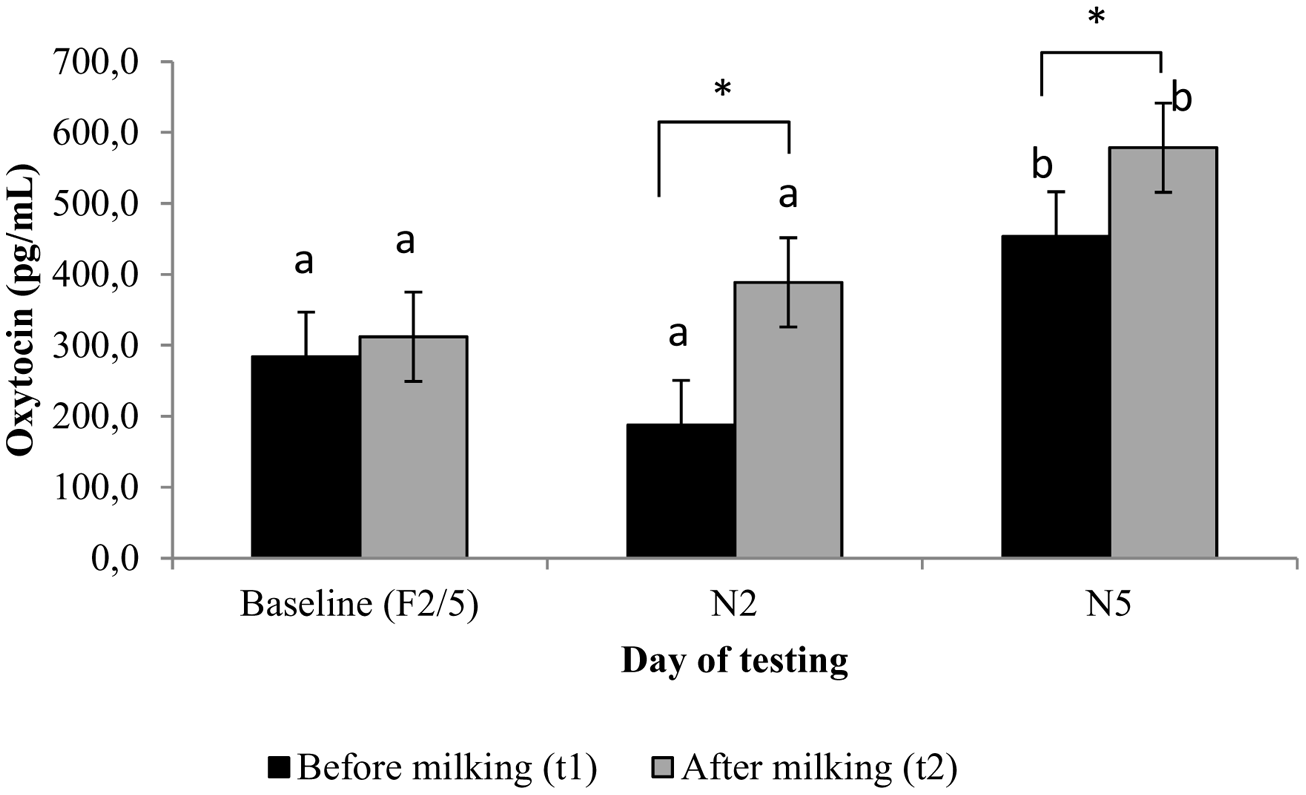
**Plasma oxytocin concentrations (pg/mL; least square means ± *SEM*) of cows (*n* = 20) before and after being milked in a familiar (Baseline) and novel milking environment (N2 and N5).** ^ab^Within sample collection time, least square means with different superscripts differ at *p* < 0.05. Means accompanied by an * differ at *p* < 0.05.

For the mean oxytocin levels at t1 and t2 on the different days, see **Figure 2**. Oxytocin levels at N2t1 tended to be lower than at F2/5t1 [*F*(2,146) = 2.61, *p* = 0.077] and were lower than at N5t1 [*F*(2,146) = 12.47, *p* = 0.0001]. Low oxytocin levels at N2t1 tended to be associated with greater behavioral response to milk cluster attachment (*r* = -0.42, *p* = 0.067) suggestive of an association with stress. Oxytocin levels at N2t1 were not related to the anticipatory cortisol response at N2t1 or the increased HR during milking on N2.

### POSSIBLE INVOLVEMENT OF OXYTOCIN IN A COPING RESPONSE TO NOVELTY

**FIGURE 3:**
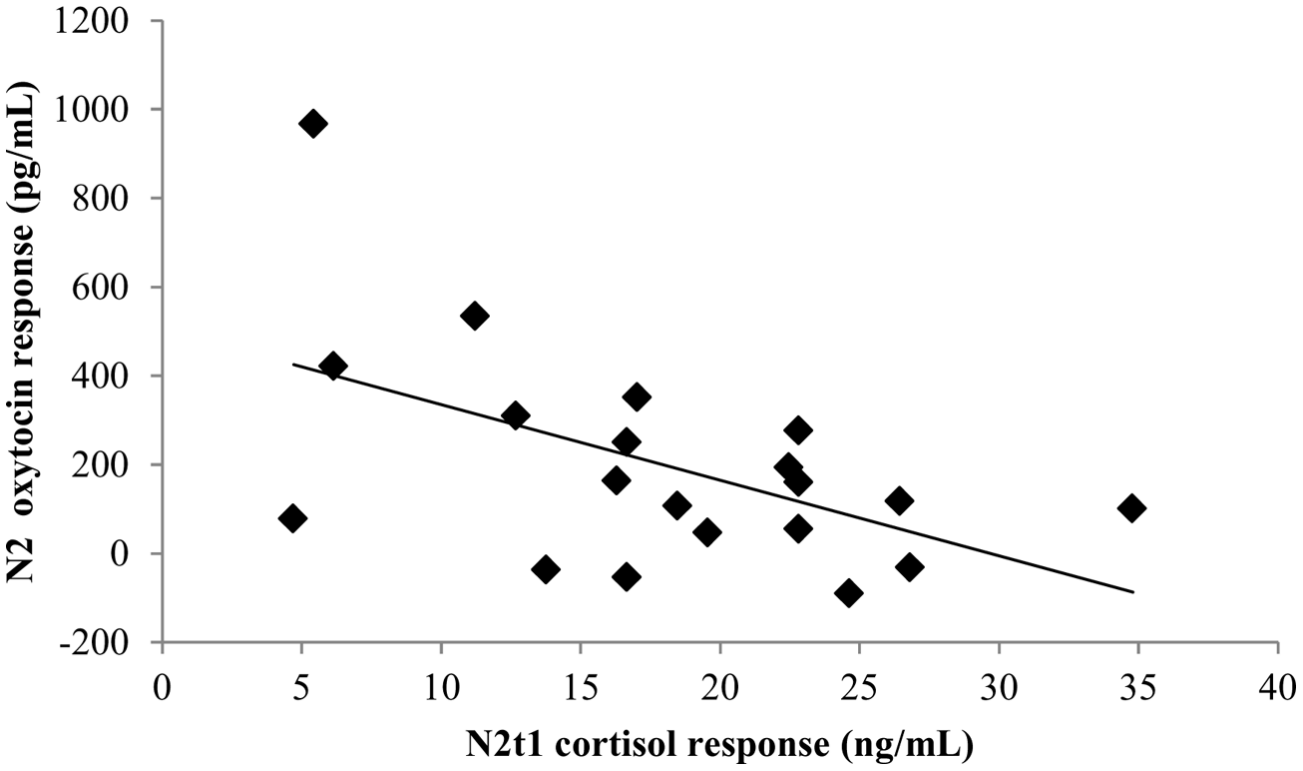
**Scatterplot of the relationship between the N2 oxytocin response to novelty and N2t1 cortisol levels**.

On N2, the oxytocin response to milking in novel conditions (t2 minus t1) was larger than on F2/5 [*F*(2,69) = 5.01, *p* = 0.010]. On N5, the oxytocin response to milking was intermediate, and did not significantly differ from F2/5 or N2. This suggests that the oxytocin response was largest in the most novel condition and smaller with increasing familiarity. The N2 oxytocin response to novelty correlated negatively with N2t1 cortisol levels (*r* = -0.50, *p* = 0.025; see **Figure 3**).

The N2 oxytocin response to novelty could not be explained by the initial low t1 oxytocin levels providing potential for a larger increase during milking. Contradicting an explanation in terms of initial values, there was a significant negative correlation between oxytocin at t1 and the increase in oxytocin levels (*r* = -0.55, *p* = 0.013) only on F2/5, the day when there was no increase during milking, whereas this correlation was not significant on N2 and N5 (*r* = -0.33 and *r* = -0.24, respectively, *p*s > 0.15). Moreover, the increase in oxytocin during milking on N2 was strongly associated with t2 levels (*r* = 0.96, *p* < 0.001).

There was reasonable reliability of individual differences in baseline (t1) oxytocin levels as indicated by correlations between days ranging from *r* = 0.51 to *r* = 0.65 (*p*s < 0.022). However, the increase in oxytocin during milking was not reliable over days, correlations between days ranging from *r* = 0.00 (between N2 and N5) to *r* = 0.19 (*p*s > 0.40). Together with the finding that oxytocin levels did not only increase during milking on N2 (especially) and N5, but also t2 levels increased from N2 to N5 [*F*(2,146) = 6.20, *p* = 0.003], the lack of reliability over days of the oxytocin increase may suggest that the cows showed individual differences in the timing of the onset and habituation of the oxytocin coping response to novelty. A proportion of the cows showed a response during milking on N2 whereas on later days an increased number of cows may have shown such a response, both in anticipation and during milking. This is supported by a high negative correlation between the oxytocin response on N2 and the increase in oxytocin from N2t2 to N5t2 (*r* = -0.58, *p* = 0.007). In other words, cows that showed a smaller increase in oxytocin on N2 showed a larger increase later on, whereas cows that showed a larger increase in oxytocin on N2 showed a smaller increase later on.

**FIGURE 4:**
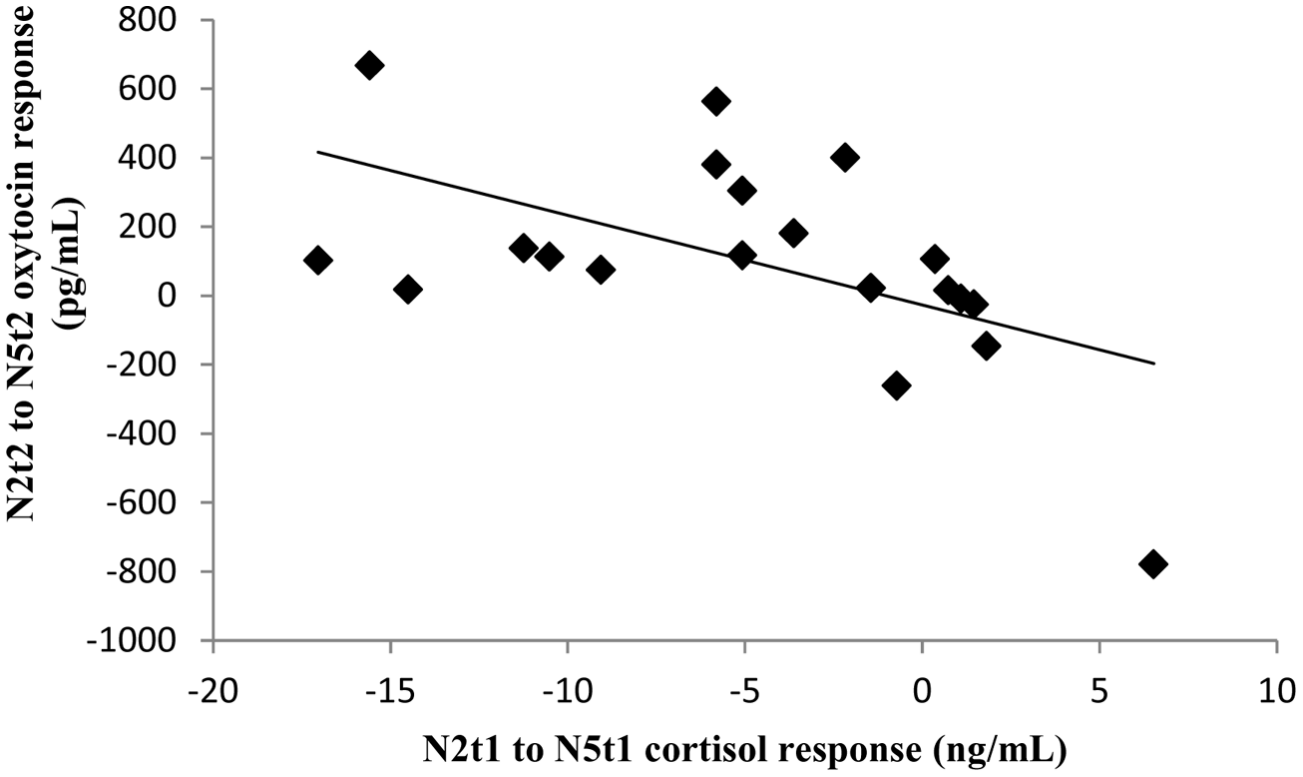
**Scatterplot of the relationship between the N5t2–N2t2 oxytocin response and the decrease in cortisol from N2t1 to N5t1**.

Crucially, if the increase in oxytocin levels from N2t2 to N5t2 reflects increased coping with the novel milking environment, then this increase in oxytocin should relate to habituation of the cortisol stress response in anticipation of milking. Indeed, this increase in oxytocin related to a decrease in cortisol from N2t1 to N5t1 (*r* = -0.50, *p* = 0.026; see **Figure 4**). We did not find correlations with HR or HRV.

## DISCUSSION

The purpose of the present study was to investigate the relationship between plasma oxytocin concentrations and the behavioral and physiological response of dairy cows to stress. In the present study, exposure to a novel milking environment was used as a psychological stressor. In previous studies, cortisol concentrations were elevated in cows in anticipation of being milked in an unfamiliar environment (Bruckmaier et al., 1993) and heart rate was elevated in primi-parous cows milked for the first time (Van Reenen et al., 2002), suggesting that exposure to a novel environment is perceived as a stressor by dairy cows. Furthermore, HRV can be used as an indicator of calmness and motivation for social behavior (Porges, 2007) and was increased by oxytocin administration in humans (Kemp et al., 2012). In the present study, cortisol levels and heart rate were elevated and HRV was lower in cows when milked in a novel environment, confirming that the cows in this study perceived the novel environment as a stressor.

On the second day of milking in the novel environment specifically, there was an increase in cortisol levels before milking that was associated with increased heart rate during milking. Moreover, a larger increase in heart rate during milking related to a larger behavioral response to cluster attachment. These changes indicate that the first encounters with the novel environment triggered a stress response that started before milking. The greater pre-milking cortisol levels observed in cows in the novel compared with the familiar environment replicates previous findings (Bruckmaier et al., 1993). We also replicated the finding of the initial suppression of oxytocin levels in a novel environment, followed by a subsequent increase (Bruckmaier et al., 1996; Macuhová et al., 2002). The decreased levels of oxytocin before milking on the second day in the novel environment coincided with increased cortisol levels. But, consistent with previous findings, those oxytocin and cortisol levels were unrelated, providing no support for a mechanism in which the increased cortisol levels suppressed the oxytocin levels (Bruckmaier, 2005). However, oxytocin levels before milking on the second day in the novel environment tended to be associated with a greater behavioral response to milk cluster attachment, suggesting a relationship between suppression of oxytocin and high fear or low trust.

Besides replicating and extending previous results from older studies showing suppression of oxytocin, there was an increase in oxytocin levels in response to novel environments (Sutherland et al., 2012). This increase was observed immediately after milking on the second and fifth day in response to been milked in a novel environment relative to oxytocin levels measured after milking in the familiar environment. Cows that showed a larger anticipatory cortisol response showed a smaller oxytocin response to novelty, suggesting that the increased cortisol levels before milking may have attenuated the oxytocin response to novelty. Moreover, studies employing more stressful conditions may not have detected the oxytocin response to novelty because of stronger attenuation of the response.

The results showed a negative association between the increase in oxytocin on the second day and the increase in oxytocin from the second to the fifth day: cows that displayed a smaller increase in oxytocin on the second day showed a larger increase later on, whereas cows that showed a larger increase in oxytocin on the second day showed a smaller increase later on. Interestingly, cortisol levels, heart rate, HRV and, non-significantly, the behavioral response to cluster attachment, showed a pattern that indicates habituation as a result of lower stress experienced by cows over time in a novel environment (i.e., indicative of stress that was highest on the second day, lower on the fifth day, and lowest on the baseline day). By contrast, only oxytocin showed a pattern of increased levels from the second to the fifth day in the novel environment and lowest levels in the familiar environment. This increase during the process of habituation is consistent with a functional role of oxytocin in habituation to a novel environment in dairy cows. Such a role of oxytocin is also consistent with our finding that, over days, the oxytocin increase was associated with habituation of the cortisol response in anticipation of milking in a novel environment.

A role of oxytocin in habituation of responses to novel environments may be important for the well-being of dairy cows. Oxytocin can influence the behavioral response to stress in animals. Oxytocin-deficient mice displayed more anxiety-related behaviors in response to an elevated plus-maze (Amico et al., 2004) and central administration of oxytocin reduced anxiety-related behaviors in rats in response to an auditory stressor (Windle et al., 1997). Moreover, a recent study in rats applying oxytocin, vasopressin and relevant antagonists, found that oxytocin promotes social proximity-seeking in response to threat (Bowen and McGregor, 2014). Oxytocin also appears involved in positive interactions between humans and animals such as gentling or petting that have been shown to have positive behavioral and physiological consequences in many species. In primates, rodents, lambs, and dogs, oxytocin has been associated with human-animal tactile contact and anti-stress effects that may influence bonding and responses to stress situations (Coulon et al., 2013). Future research investigating the relationship between the behavior of handlers and of familiarity of the cows with the handler on stress regulation in dairy cows would be of interest. Moreover, a mechanism of oxytocin-modulated habituation of stress responses may be important in humans as well (Tops et al., 2013).

Limitations of this study include the small number of cows and the partly correlational nature of our results. The correlational data we presented do not allow conclusions about a functional role of oxytocin in stress habituation processes or about the direction of causality, but lays the foundations for further investigation of the role of oxytocin and its interrelation with the levels of cortisol in response to psychological stressors. Moreover, we did not find significant effects of novelty on our behavioral measure, the FSK score. Sutherland and Dowling (2014) found that the FSK score of heifers was reduced by almost 50% by the sixth week of lactation, suggesting that the performance of FSK behaviors during the first week of lactation is most likely related to fear of humans and/or the novel milking environment. However, Bremner (1997) found that the response of heifers to milk cluster attachment (e.g., FSK) during the first week of lactation was non-linearly related to the amount of handling they received prior to calving. For example, heifers that received the least amount of handling and heifers that received the most amount of handling prior to calving moved and kicked less during the milking process. These findings suggest that both tame and fearful animals move and kick less during milk cluster attachment. Future studies should include additional behavioral measures besides FSK.

In conclusion, the results from this study suggest that oxytocin release is increased in response to exposure to a psychological stressor (novel environment) and that cows adapt to this stressor over time. After initial suppression, oxytocin levels increased over days of milking in a novel environment. Furthermore, the oxytocin increase was associated with habituation of the cortisol response in anticipation of milking in a novel environment, suggesting that oxytocin may be involved in habituation to a novel environment in dairy cows. Therefore, cows that have a greater oxytocin response to a psychological stressor may cope better in response to psychological stressors.

## Conflict of Interest Statement

The authors declare that the research was conducted in the absence of any commercial or financial relationships that could be construed as a potential conflict of interest.
